# Supplementary material for: Asymmetric Synthesis of Quaternary Hydantoins via a Palladium-Catalyzed Aza-Heck Cyclization
Source: J Am Chem Soc. 2025 Nov 14;147(49):44692–8. doi: 10.1021/jacs.5c16022 (PMC12703750; doi:10.1021/jacs.5c16022)
Supplement: Supplementary file 2 [file ja5c16022_si_002.zip › All NMR FID Files/S8 After Heating/S8-A_CarbonNMR/7/pdata/1/email_EM06-035-isomer#1-13C_7_1.pdf]

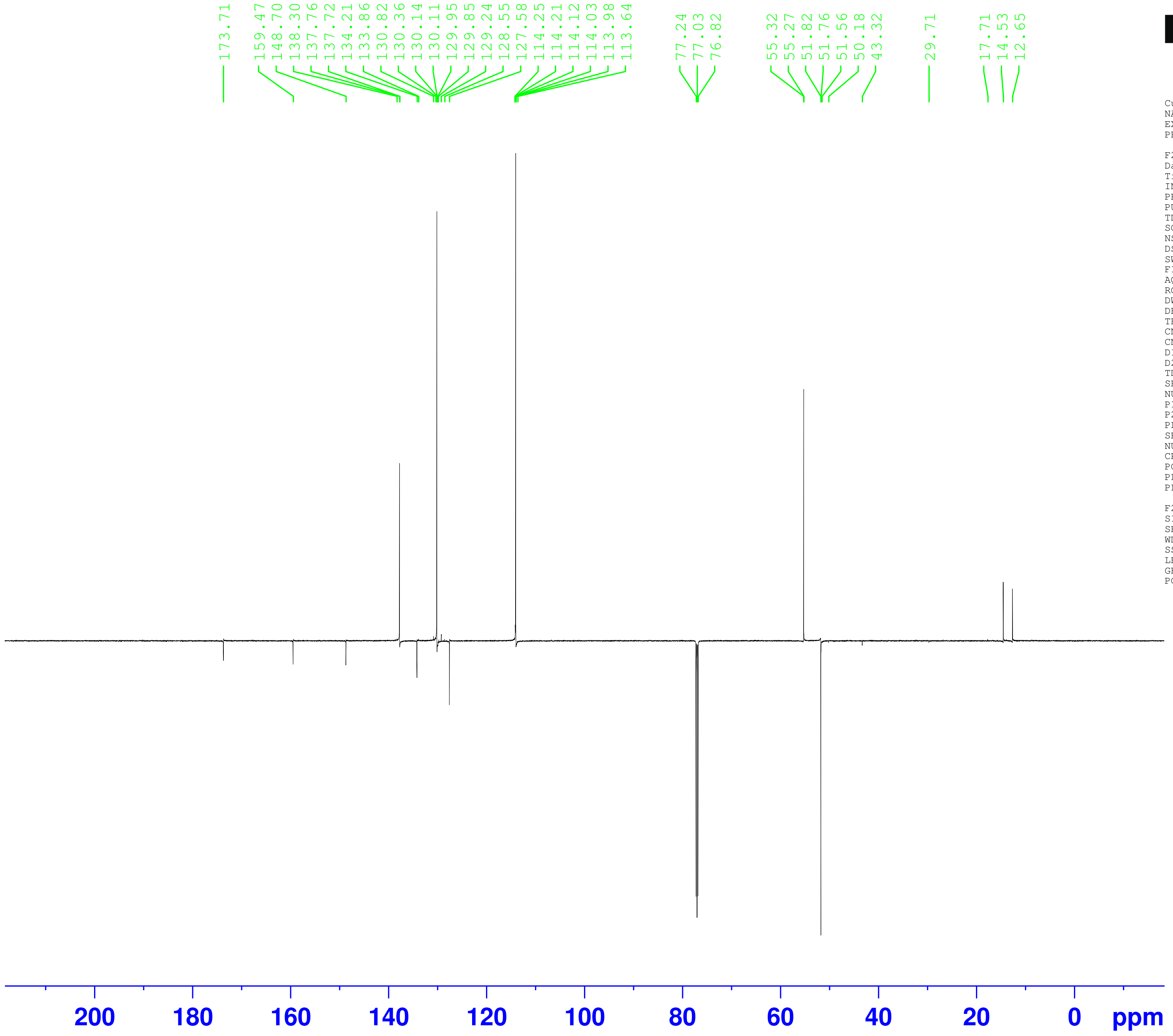


```
Current Data Parameters
NAME       EM06-035-isomer#1-13C
EXPNO                          7
PROCNO                         1

F2 - Acquisition Parameters
Date_                   20250211
Time                     23.44 h
INSTRUM    Avance Neo 600 at University of Delaware
PROBHD     Z185170_0001 (
PULPROG                     jmod
TD                         65536
SOLVENT                    CDCl3
NS                          2048
DS                             4
SWH                    35714.286 Hz
FIDRES                  1.089913 Hz
AQ                     0.9175040 sec
RG                           101
DW                        14.000 usec
DE                         19.25 usec
TE                         298.0 K
CNST2                145.0000000
CNST11                 1.0000000
D1                    2.00000000 sec
D20                   0.00689655 sec
TD0                            1
SFO1                 150.9178988 MHz
NUC1                         13C
P1                         11.98 usec
P2                         23.96 usec
PLW1                106.00000000 W
SFO2                 600.1324005 MHz
NUC2                          1H
CPDPRG[2                 waltz65
PCPD2                      70.00 usec
PLW2                  6.90000010 W
PLW12                 0.09170600 W

F2 - Processing parameters
SI                         32768
SF                   150.9028085 MHz
WDW                           EM
SSB                            0
LB                          1.00 Hz
GB                             0
PC                          1.40
```
